# Correction: Alfei et al. 4-Hydroxybenzoic Acid as an Antiviral Product from Alkaline Autoxidation of Catechinic Acid: A Fact to Be Reviewed. *Plants* 2022, *11*, 1822

**DOI:** 10.3390/plants12081635

**Published:** 2023-04-13

**Authors:** Silvana Alfei, Debora Caviglia, Susanna Penco, Guendalina Zuccari, Fabio Gosetti

**Affiliations:** 1 Department of Pharmacy (DIFAR), University of Genoa, Viale Cembrano, 4-16148 Genoa, Italy; 2 Department of Surgical Sciences and Integrated Diagnostics (DISC), University of Genoa, Viale Benedetto XV, 6-16132 Genoa, Italy; 3 Department of Experimental Medicine, University of Genoa, Via Leon Battista Alberti, 2-16132 Genoa, Italy; 4 Department of Earth and Environmental Sciences (DISAT), University of Milano-Bicocca, Piazza della Scienza, 1-20126 Milano, Italy

## Error in Figure

In the original publication [1], there was a mistake in Figure 5 as published. CD_3_OD was written in place of HDO near the peak at about 4.8 ppm on the ^1^H NMR spectrum of 4-HBA. The corrected Figure 5 appears below.


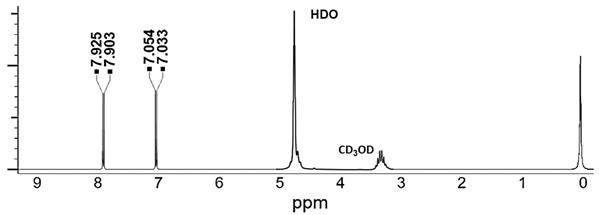


Figure 6 was published with horizontal orientation instead of vertical one. The graph has also been corrected in the original publication.

In Figure S3 of the Supplementary Materials, CD_3_OD was written in place of HDO near the peak at about 4.8 ppm on the ^1^H NMR spectrum of catechinic acid (CA). The corrected Figure S3 appears below.


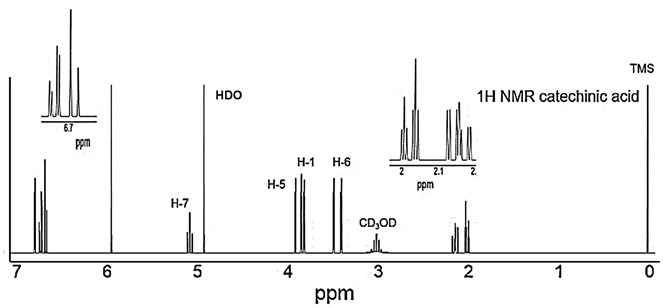


## Text Correction

There were some language/expression issues in original publication. Excessive information or personal comments not necessary to understand the reported study were included in the original publication.

A correction has been made to Abstract (Page 1):

The findings seemed not to be reliable because of the existence in the literature of very different findings, because of the high concentration that was attributed to the supposed 4-HBA in the dark mixture, and because of the absence of essential analytical experiments to confirm 4-HBA in AOCA. Particularly, the AOCA chromatograms highlighting a peak attributable to 4-HBA, using commercial 4-HBA as a standard, is missing, as well as investigations concerning the antiviral activity of marketed 4-HBA.

A correction has been made to Introduction, Paragraph 4 (Page 2):

In a recent study, the main constituent (75%) of AOCA was isolated and named compound **2** [8]. It was identified as the colorless and well-known 4-hydroxy benzoic acid (4-HBA), and it was reported to be responsible for the antiviral activity of AOCA, as **2** had been found to be active on tomato brown rugose fruit virus (ToBRFV). This finding turned out to be very different from what was already widely reported in several specific articles [9–13]. In this regard, the structural identity recently attributed to **2** was validated, since 4-HBA is commonly produced by the thermolytic degradation of some flavonoids [14,15]. However, the thermolytic conditions under which this occurs are considerably more drastic than those used to produce AOCA. Moreover, it is known in the literature that 4-HBA derives only from flavonoids structurally different from catechin [14,15]. In particular, catechin, encompassing two catechol phenyl rings both having two hydroxyl groups, it is unlikely to thermolytically provide 4-HBA, which possesses only one hydroxyl group. In fact, it is reported that 4-HBA derives from flavonoids with at least one phenyl ring having only one hydroxyl function, and whose structure can precisely degrade to 4-HBA [14,15].

A correction has been made to Introduction, Paragraph 4 (Page 3):

Additionally, the other compounds described by Laks et al. [10], Ohara et al. [11], and Hashida et al. [12,13], as originating from the alkaline degradation of CA, recently were not detected together with compound **2** [8]. Anyway, we think that a simple experiment to confirm the structural identity (4-HBA) attributed to **2** could have been performed, in the form of testing a commercial sample of 4-HBA on ToBRFV, confirming or refuting its identical antiviral activity. Precisely for this purpose, we tested, as reported by Ferrea et al. in 1993, a commercial sample of 4-HBA on VERO cells infected with HSV-2 viruses and discovered that 4HBA was completely inactive. Therefore, also for this reason, the identity attributed to compound **2**, found active against ToBRFV, was unlikely, especially at the high concentration reported (75%). In addition, a chromatogram of AOCA that highlighted the presence of a peak attributable to 4-HBA, using a commercial sample of 4-HBA as a reference compound, has not been provided [8]. Therefore, we undertook this study to verify the accuracy of the identity attributed to the molecule (**2**) that the authors were able to separate [8]. 

A correction has been made to Introduction, Final Paragraph (Page 3):

Finally, the antiviral effects of AOCA against HSV-2 were investigated and confirmed, thus establishing that the AOCA prepared by us, and not containing 4-HBA, is identical to the one recently reported and testified to contain 4-HBA, but whose activity against HSV-2 had not been verified.

A correction has been made to Results and Discussion, Section 2.1, Paragraph 1 (Page 3):

CA was prepared as previously described [5,7,8,13], starting from commercial (+)-catechin according to Scheme 1, in which the numbering of atoms of catechin and CA has been reported, to facilitate an understanding of the mechanism of the reaction and to clarify the assignments reported in the peak lists of NMR analyses (Materials and Methods section). We make note that the correct structure of CA presents the catechol ring linked to C-6 and not to C-8 as recently reported [8]. In fact, C8 should have two diastereotopic proton atoms, i.e., Ha and Hb and not only one, proton atom, just as herein observable in Figure 1 and Scheme 1.

A correction has been made to Results and Discussion, Section 2.1, Page 4, before Scheme 1: 

Notably, extremely neat CA was achieved, without recovery via high-cost and long-term semi-preparative HPLC, which uses large amounts of solvents and costly apparatus, as recently reported [8]. In our opinion, special purification techniques should be used when strictly necessary to lower costs and respect the environment. The structure of the obtained CA, which we isolated in a simple, cheap, and fast manner, was confirmed by ATR–FTIR (Figure 2) and ^1^H and ^13^C NMR spectroscopy (Figures S3 and S4 in Section S1 of Supplementary Materials), while its purity was assessed first by TLC (Figure S1, Section S1, Supplementary Materials), and then by UV–Vis and UHPLC–MS/MS analyses (Figures 3 and 4), and finally further confirmed by elemental analyses.

A correction has been made to Results and Discussion, Section 2.1, Page 5, sentence below Figure 3:

The results obtained by us from UV–Vis and ATR–FTIR analyses conformed to those reported by Sears et al. (UV–Vis and FTIR) [5] and Kennedy et al. (UV–Vis) [18].

A correction has been made to Results and Discussion, Section 2.2, Pages 6–7:

Starting from CA, we prepared AOCA by reproducing exactly the protocol developed in the past by Ferrea et al. [5] and recently reproduced [8]. AOCA was obtained as a dark brown solid, as observable in Figure S2 (Section S1 Supplementary Materials). Additionally, we completely characterized AOCA in terms of physicochemical analyses, by TLC, ATR–FTIR, UV–Vis and UHPLC–MS/MS, which were not carried out so far.

In particular, we prepared AOCA and carefully analyzed it by several analytical techniques to unequivocally verify the accuracy of the structural identity attributed to compound **2** (identified as 4-HBA) recently isolated as the main constituent of AOCA and reported as responsible for its good antiviral activity [8]. However, compound **2** has been shown to be active on a completely different type of virus (i.e., on ToBRa, a naked RNA virus) compared to the one on which the AOCA was originally found to be active (i.e., HSV-1 and HSV-2, DNA viruses, coated with an envelope) [5]. This study was motivated by the lack of literature reporting 4-HBA as a constituent, at least in trace amounts, of the products of the alkaline oxidation of catechin or CA [9–13], and by the presence of several literature studies that describe the remarkable stability of the CA nucleus under the alkaline oxidative conditions [16]. Moreover, since we believed that analyses such as HPLC or at least TLC, highlighting the actual presence of 4-HBA in AOCA, were necessary, they were herein carried out by us. Furthermore, the results from NMR analysis carried out on compound **2** are not totally in accordance with those known for commercial samples of 4-HBA. In particular, even if, in the ^1^H NMR spectrum of **2**, signals compatible with the AA’XX’ system of the p-disubstituted phenyl ring of 4-HBA are observable at 7.87 ppm (d, 2H, J = 8.00 Hz, CH (2/6)) and 6.82 ppm (d, 2H, J = 8.01 Hz, CH (3/5)), other, not negligible signals are present at 0.5–1.5 ppm (particularly 0.8 and 1.5 ppm), which are missing in the spectrum of a commercial sample of 4-HBA (Figure 5).

A correction has been made to Results and Discussion, Section 2.2, Paragraphs below Figure 5, Page 7: 

For the precise assignment of the signals of the AA’XX’ system observable in the ^1^H NMR spectrum of **2**, HSQC and HMBC 2D experiments have been reported [8]. In an HSQC spectrum, a ^13^C spectrum is displayed on one axis and a ^1^H spectrum is displayed on the other axis, and cross-peaks show which proton is attached to which carbon considering only C-H (only one-bond coupling). Meanwhile, two-, three-, or sometimes even four-bond couplings are signaled in an HMBC spectrum. In particular, H-C-C or H-C-C-C, or even H-C-C-C-C, and not H-C, are visible. Regarding this, in the HSQC spectrum of **2**, one-bond couplings between the proton atom(s) at 1.5 ppm with carbon(s) having signals in the range 20–40 ppm are visible, which are not compatible with the structure of 4-HBA [8]. Furthermore, since it was assumed that **2** could be 4-HBA, the ^1^H NMR spectrum was acquired in deutero methanol (which does not allow one to see the signals of exchangeable protons, thus losing valuable information) rather than in deutero dimethyl sulfoxide. The dimethyl sulfoxide spectrum would have instead allowed to confirm or refute the presence of the COOH and OH groups of 4-HBA.

Finally, differently from **2** (identified as 4-HBA) [8], a sample of commercial 4-HBA tested by us on VERO cells infected by HSV-2 was not active.

Based on these considerations, to verify the actual presence of 4-HBA among AOCA constituents, we examined the AOCA prepared by us, performing different analytical techniques essential to confirm the identity assigned to **2**. In the following section of this article, the performed analyses, the results, and a brief discussion are given.

A correction has been made to Results and Discussion, Section 2.2.1, Last Paragraphs below Figure 6, Page 8:

As observable in both Figure S5 (254 nm) and Figure 6a (365 nm), there is no trace in AOCA of a spot corresponding to the Rf of 4-HBA, thus indicating the absence of 4-HBA in AOCA.

A correction has been made to Results and Discussion, Section 2.2.2, First sentence below Figure 7, Page 9: 

As Figures 7 and S6 show, in the AOCA spectrum (λ_max_ = 268 nm), no peak is detectable at λ_max_ = 257 nm, corresponding to 4-HBA absorbance, while, considering the recently reported high concentration of **2** in AOCA (75% *wt*/*wt*), if this had been the case, a significant absorption at λ_max_ = 257 nm should have been observed in AOCA.

A correction has been made to Results and Discussion, Section 2.3, First Paragraph, Page 12: 

To verify that the AOCA prepared by us, and that of Ferrea et al. [5], who found AOCA active against HSV-1 and HSV-2, were comparable, thus validating our findings, we repeated, with AOCA on VERO cells the same tests carried out by Ferrea. In addition, hoping to highlight an antiviral activity of 4-HBA right on HSV-2, the same experiments were conducted with a commercial sample of 4-HBA.

A correction has been made to Conclusions, Page 15: 

Compound **2** was recently isolated from the product of the oxidation of Combretum leaf extracts and catechinic acid (CA), namely AOCA. It was discovered to be active against ToBRFV and was identified as 4-HBA. Here, an unusual study has been reported, aimed at verifying the presence of 4-HBA among the constituents of AOCA (at a concentration above 50%) and its actual antiviral activity. To this end, we have prepared CA from (+)-catechin of plant origin and in turn we have prepared AOCA from CA. AOCA has been subsequently analyzed by performing TLC, UV–Vis, UHPLC–MS/MS, and chemometrically assisted ATR–FTIR experiments, unequivocally ascertaining that 4-HBA is not a constituent of AOCA. Differently from AOCA prepared by us and AOCA prepared by Ferrea et al., a commercial sample of 4-HBA was completely inactive when tested on HSV-2. From results of our study the identity of compound **2** remains unknown. With this work we have evidenced that the materials deriving from plants extraction are very complex mixtures of compounds, difficult to be separated and identified, so that detailed studies and verifications are necessary, as well as the development of new analytical methods.

The authors state that the scientific conclusions are unaffected. This correction was approved by the Academic Editor. The original publication has also been updated.
