# Supplementary material for: 4-Hydroxybenzoic Acid as an Antiviral Product from Alkaline Autoxidation of Catechinic Acid: A Fact to Be Reviewed
Source: Plants (Basel). 2022 Jul 11;11(14):1822. doi: 10.3390/plants11141822 (PMC9325093; doi:10.3390/plants11141822)
Supplement: Supplementary file 1 [file plants-11-01822-s001.zip › plants-1815103-supplementary.pdf]

Supplementary Materials

4-Hydroxybenzoic Acid as an Antiviral Product from Alkaline Autoxidation of Catechinic Acid: A Fact to Be Reviewed

Silvana Alfei [1,*], Debora Caviglia [2,*], Susanna Penco [3], Guendalina Zuccari [1] and Fabio Gosetti [4]

1. Department of Pharmacy (DIFAR), University of Genoa, Viale Cembrano, 4 - 16148 Genoa, Italy; zuccari@difar.unige.it (G.Z.)
2. Department of Surgical Sciences and Integrated Diagnostics (DISC), University of Genoa, Viale Benedetto XV, 6, 16132 Genoa, Italy
3. Department of Experimental Medicine, University of Genoa, Via Leon Battista Alberti, 2 - 16132 Genoa (GE), Italy; susanna.penco@unige.it (S.P.)
4. Department of Earth and Environmental Sciences (DISAT), University of Milano-Bicocca, Piazza della Scienza, 1 - 20126 Milano, Italy; fabio.gosetti@unimib.it (F.G.)

* Correspondence: alfei@difar.unige.it; Tel.: +39 010 355 2296 (S.A.); debora.caviglia@edu.unige.it (D.C.)

Table S1. SRM NI transitions (Q1 and Q3 masses), dwell time, retention time (t_R), and mass spectrometry parameters: Q1 Pre Bias, CE (Collision Energy), Q3 Pre Bias. The most three sensitive transitions for each species were monitored.

Analytes	Q1 (*m/z*)	Q3 (*m/z*)	Dwell time (ms)	t_R (min)	Q1 pre Bias (V)	CE (V)	Q3 pre Bias (V)
CA	289	245/109/203	43	0.87	11/30/15	15/19/27	11/20/10
4-HBA	137	93/65/137	43	1.86	10/10/10	20/27/10	13/11/14
(+)-catechin	289	245/109/203	43	1.98	14/16/11	16/24/19	11/10/13

Section S1. (+)-Catechin and Catechinic Acid (CA)

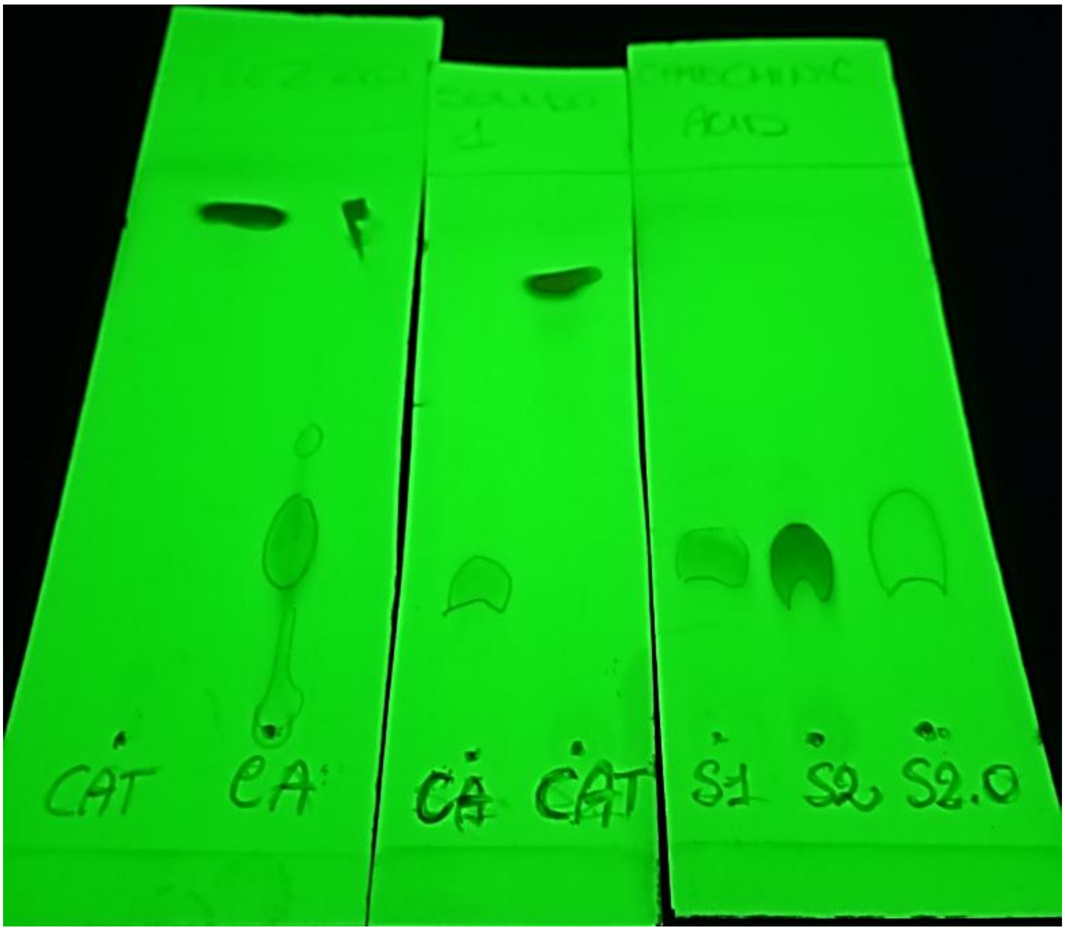

Figure S1. TLCs image observed at 254 nm (eluent ethyl acetate (EtOAc)/methanol (MeOH)/acetic acid (AcOH) 9/1/0.2 v/v/v). From the left: (+)-catechin (Rf. 0.82) and crude CA (Rf. 0.26); isolated CA and (+)-catechin; three samples of CA obtained by three different crystallizations from acetone/ethyl ether.

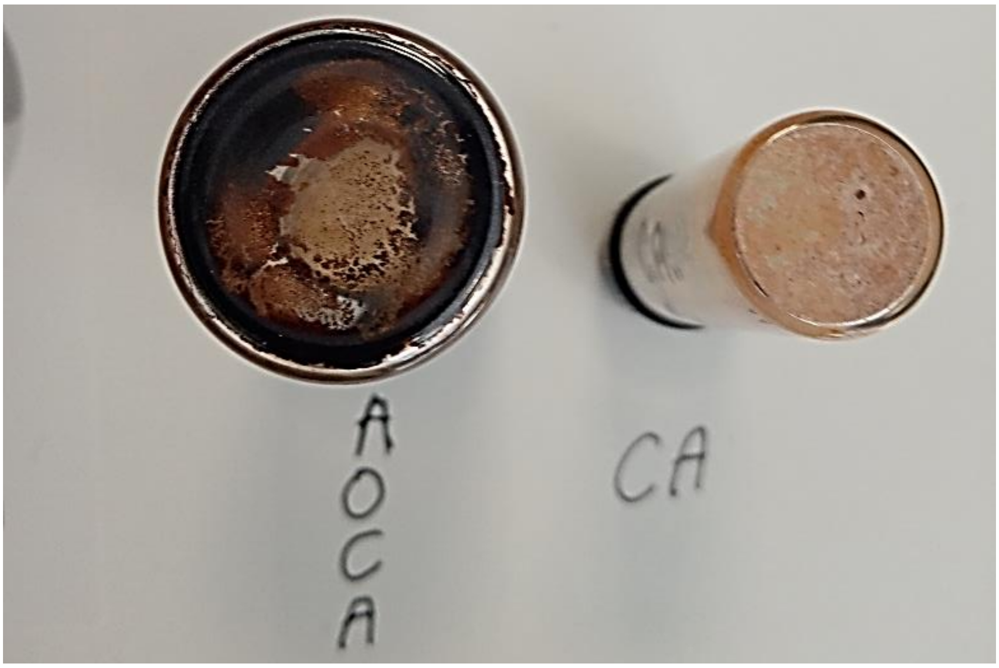

Figure S2. Aspect of the crystallized CA (right) and of the product of alkaline autooxidation of CA (AOCA).

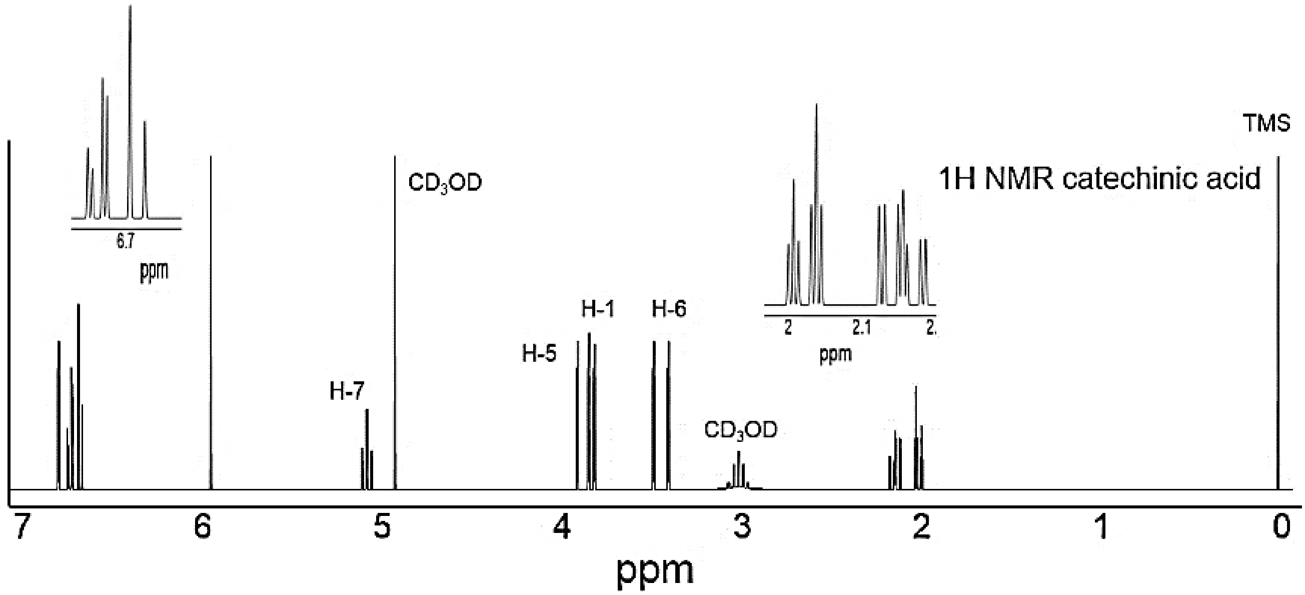

Figure S3. ^{1}H NMR (CD_3OD, 400 MHz) spectrum of CA.

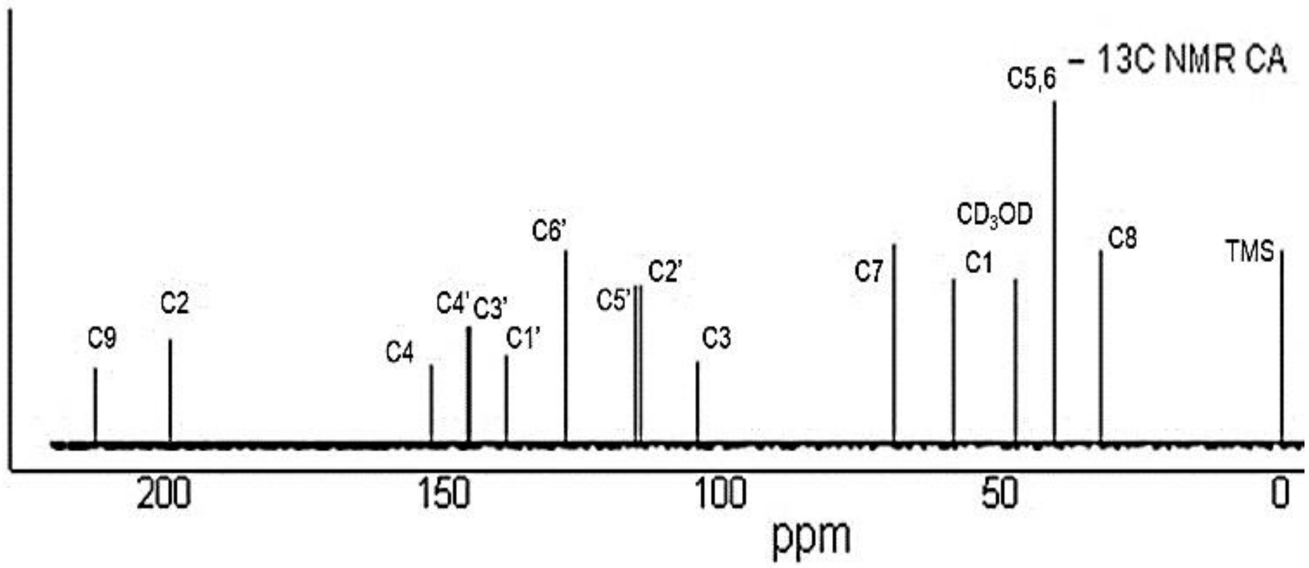

Figure S4. ^{13}C NMR (CD_3OD, 100 MHz) spectrum of CA.

Section S2. AOCA vs 4-HBA

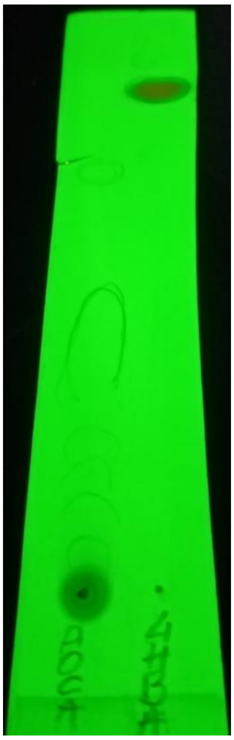

Figure S5. TLC analysis on AOCA and 4-HBA, taken as reference compound (detection at 254 nm) showing the absence of 4-HBA in AOCA.

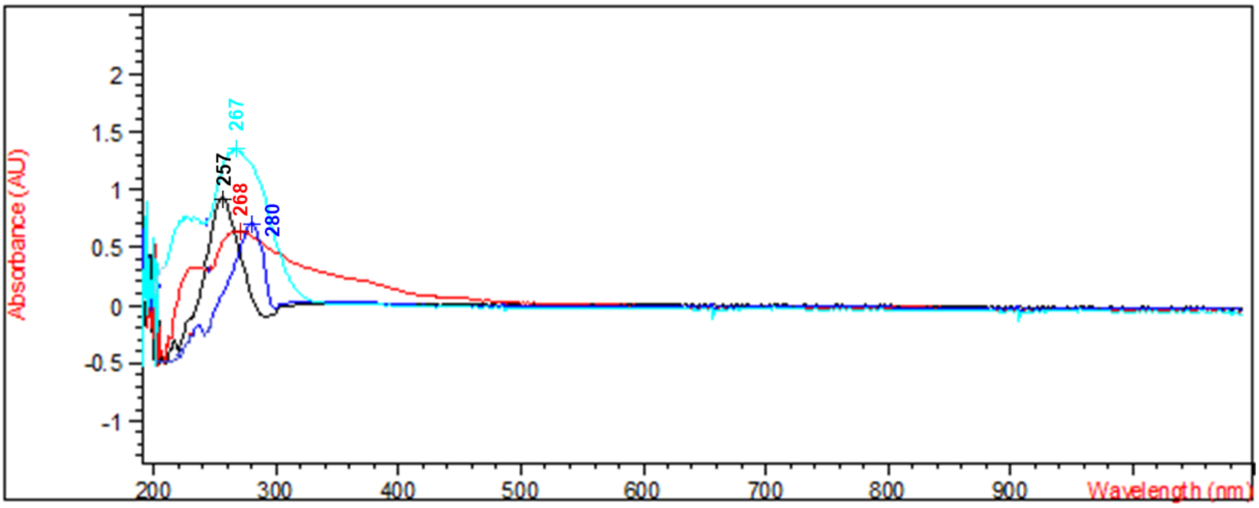

Figure S6. UV-Vis spectra of (+)-catechin (blue line), CA (light-blue line), AOCA (red line) and 4-HBA (black line).

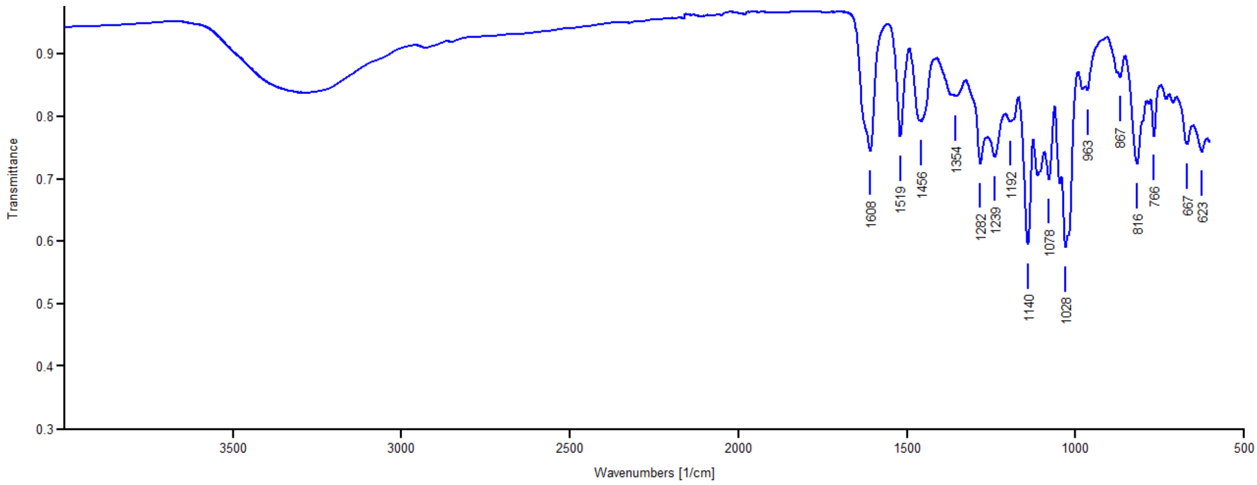

Figure S7. ATR-TFIR spectrum of (+)-catechin.

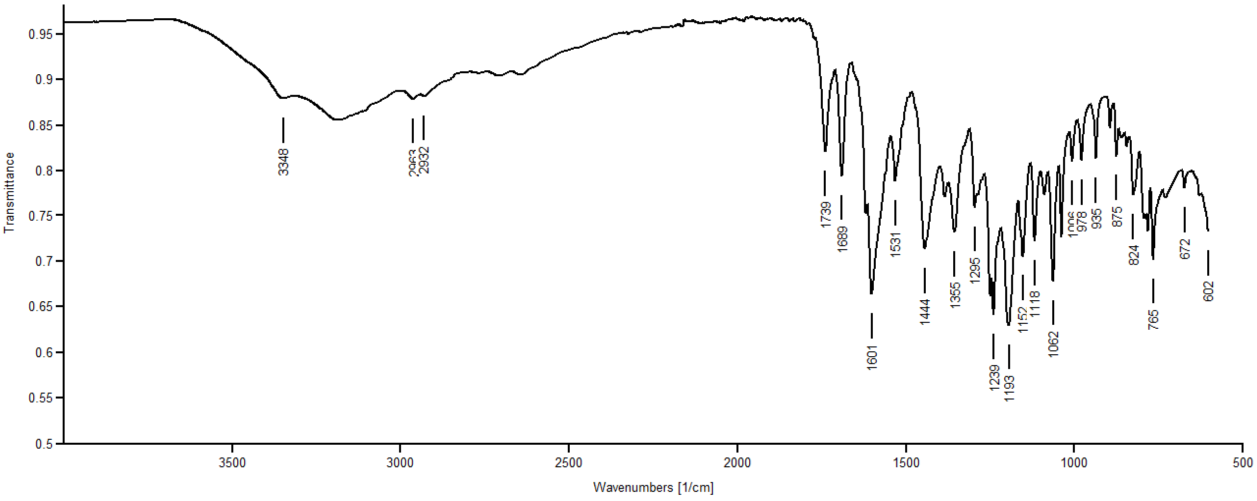

Figure S8. ATR-TFIR spectrum of CA.

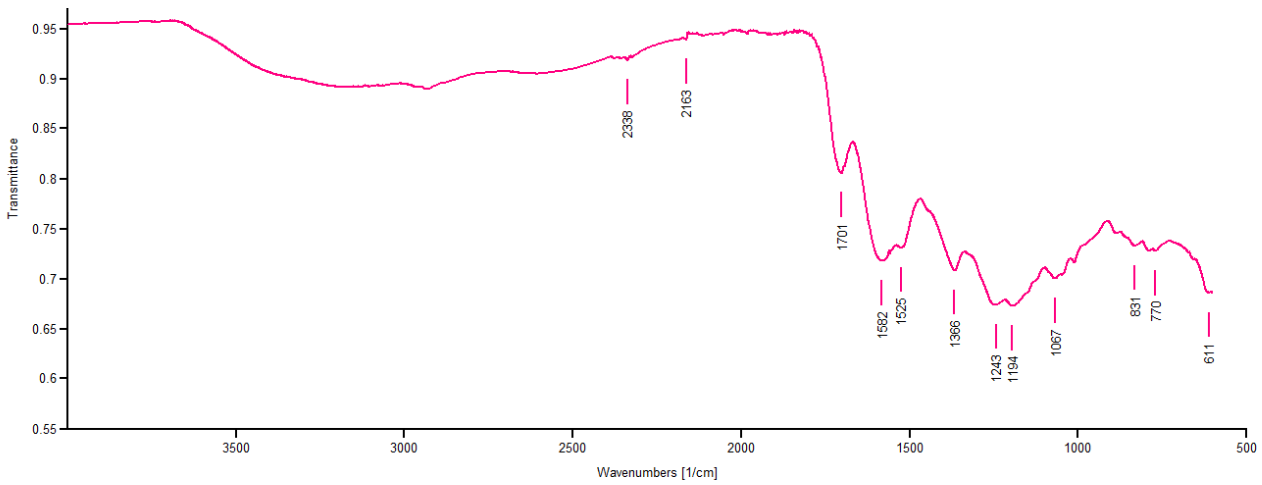

Figure S9. ATR-TFIR spectrum of AOCA.

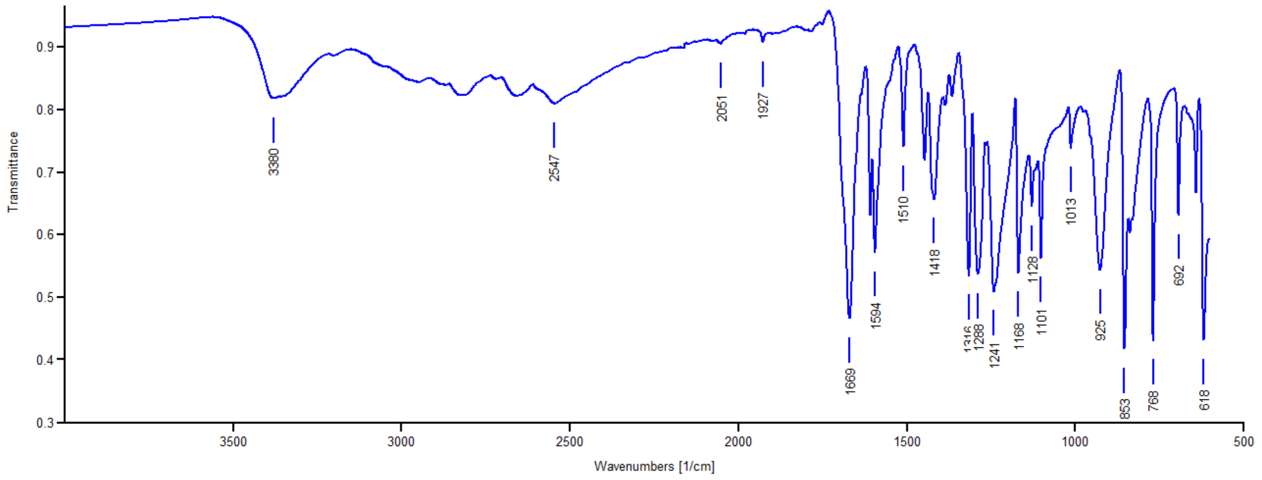

Figure S10.ATR-TFIR spectrum of 4-HBA.
